# Estimating the impact of neonatal abstinence system interventions on Medicaid: an incremental cost analysis

**DOI:** 10.1186/s13011-021-00427-1

**Published:** 2021-12-20

**Authors:** Diana López-Soto, Paul M. Griffin

**Affiliations:** 1 Department of Industrial Engineering, Tecnológico de Monterrey, Guadalajara, Mexico; 2 Department of Industrial Engineering, Penn State University, University Park, PA USA

**Keywords:** Neonatal abstinence syndrome, Treatment interventions, Incremental cost analysis

## Abstract

**Background:**

Neonatal abstinence syndrome (NAS) incidence has significantly increased in the US in recent years. It is therefore important to develop effective intervention protocols that mitigate the long-term consequences of this condition for the mother, her child, and the community.

**Methods:**

We used Monte Carlo simulation to estimate the impact of four interventions for NAS and their combinations on pregnant women with opioid use disorder. The key outputs were changes in incremental costs from baseline from the Medicaid perspective and from a total systems perspective and effect size changes. Simulation parameters and costs were based on the literature and baseline model validation was performed using Medicaid claims for Indiana.

**Results:**

Compared to baseline, the resulting simulation estimates showed that three interventions significantly decreased Medicaid incremental costs by 8% (mandatory opioid testing (MOT)), 4% (patient navigators), and 3% (peer recovery coaches). The combination of the three interventions reduced Medicaid direct costs by 26%. Reductions were similar for total system incremental costs (ranging from 2 to 24%), though MOT was found to increase costs of overdose death based on productivity loss. NAS case reductions ranged from 1% (capacity change) to 13% (MOT).

**Conclusions:**

Using systems-based modeling, we showed that costs associated with NAS can be significantly reduced. However, effective implementation would require the involvement and coordination of several stakeholders. In addition, careful protocols for MOT should be considered to ensure pregnant women don’t forgo prenatal care for fear of punitive consequences.

**Supplementary Information:**

The online version contains supplementary material available at 10.1186/s13011-021-00427-1.

## Background

The average US neonatal abstinence syndrome (NAS) incidence rose from 1.6 per 1000 in-hospital births in 2004 to 8.8 per 1000 births in 2016 [1] with Medicaid covering 82% of those births in 2014 [2]. Hospital charges due to NAS in the US rose from $190 M in 2000 to $720 M in 2009 [3]. In addition, for the dyad of mothers with NAS and their infants, maternal mortality is 6.4 times higher and neonatal mortality and/or severe mortality rates are 3.7 times higher than the corresponding rates of dyads without NAS [4].

A variety of evidence-based non-pharmacologic interventions have been developed for NAS including rooming-in with family [5, 6], breast feeding [6, 7], and kangaroo care [8]. Effectively addressing NAS is difficult, however, due to required involvement and coordination of multiple stakeholders including pediatric, neonatal intensive care, OBGyN, and nursery units in the hospital along with treatment clinics and social services [9], and typically requires a portfolio of interventions to be impactful [9, 10].

We estimated the incremental cost impact of four commonly available interventions and their combinations compared to baseline for opioid based NAS on both Medicaid and overall system costs using Monte Carlo simulation. The interventions considered were: i) mandatory opioid testing (MOT) (a urine test given during prenatal care), ii) the use of peer recovery coaches (individuals that successfully completed long term treatment are used to engage the woman through their treatment program), iii) capacity increases (through the removal of treatment barriers), and iv) patient navigators (help guide women through enrollment into services and provide transportation). The results can help provide guidance to providers for intervention investment and to state Medicaid policymakers as to which interventions (or combinations) are beneficial to cover.

## Methods

We developed a Monte Carlo simulation model representing the US population of Medicaid-enrolled women with opioid use disorder (OUD) and their resulting pathways. This population was chosen since it represents the majority of resulting NAS cases. For each simulation run, each woman goes through a probability tree of sequential stages including pregnancy, treatment, and outcomes with associated costs. Online Appendix 1 shows the probability trees for each of the study instances and the parameters and sources used in the simulation. Note that although there is a cumulative effect from multiple interventions, because they can interact with each other, the output is not the same as the sum of the interventions modeled in the scenario.

Costs considered in the analysis were daily hospital cost, daily NAS treatment cost, intervention costs, special education costs for children with NAS, treatment costs for the mother (methadone, buprenorphine, detoxification, psychological support), and annual incremental healthcare costs resulting from OUD. Death due to overdose was quantified based on the present value of lifetime productivity by age. Cost details are provided in Online Appendix 2. Note that all costs considered were incremental due to NAS. The study followed the CHEERS checklist for economic evaluations of health interventions.

The key outcome from the simulation was the average total cost (and standard error) for the population of pregnant women. We considered it from two perspectives. The first perspective was based on direct Medicaid costs of billable treatment for the mother and child and the intervention costs. The second perspective added the costs of overdose death, living with OUD, and special education for children both with NAS for overall system costs. We compared the interventions and their combination to the baseline case of no intervention based on average cost. As the time horizon was one year, costs were not discounted. We also determined the effect size in terms of number of pregnant women compared to baseline for those starting treatement, those completing treatement, and NAS cases for each of the interventions and their combinations.

For each scenario, including baseline and 12 intervention combinations, we ran the simulation for one year of time using 500 replications of 200,000 women with OUD. This was sufficiently large to ensure stability of the sample means for the outputs of interest.

For model validation, we compared the outcomes of interest for a cohort of pregnant women generated under no intervention (i.e., baseline) to estimates found from Medicaid claims for the state of Indiana in 2017–2018. For the number of pregnant women that started treatment, we were not able to find appropriate estimates in the literature. We therefore determined from all women that delivered in Indiana in 2018, those that had any ICD10 code for OUD within 1 year of their delivery date. Among this group we then found those women who had an ICD10 code for any type of treatment (detoxification or MAT).

## Results

Table 1 shows the simulation results for direct Medicaid incremental costs. Note that for this population, there was an average of 7925.4 pregnant women (SD = 80.7) at baseline, with total Medicaid costs of $631,986 (SD = $18,367). All interventions other than capacity expansion alone led to a significant percentage decrease in average total cost, ranging from 3 to 26%. The single intervention with the greatest percentage cost decrease compared to baseline was MOT (8%) and the intervention combination with the greatest percentage decrease was the combination of MOT with peer recovery coaching and peer navigators (26%).

**Table 1:** Average Medicaid incremental cost differences compared to baseline for four interventions and their combinations (all differences are significant unless shown otherwise)

Intervention	Medicaid Incremental Costs ($)			Percent Difference from Baseline
	Opioid Treatment for Mother	NAS Treatment	Total (95% CI)	
Baseline	5004	266,644	271,648 (270,756, 272,540)	
MOT	15,972	234,447	250,419 (249,527, 251,311)	−8%
Patient Navigators	9768	252,321	262,089 (261,197, 262,981)	−4%
Capacity Increase	5963	264,207	270,170 (269,277, 271,062)	−1% (NS)
Peer Recovery Coaches	5232	256,963	262,195 (261,302, 263,086)	−3%
Navigators + Coaches	10,229	233,914	244,143 (243,250, 245,035)	−10%
MOT + Navigators	20,783	221,061	241,844 (240,951, 242,736)	−11%
MOT + Capacity	16,960	230,935	247,895 (247,002, 248,786)	−9%
MOT + Coaches	16,789	204,083	220,872 (219,980, 221,764)	−19%
MOT *+* Navigators + Coaches	21,789	179,423	201,212 (200,320, 202,104)	−26%
Navigators + Capacity	10,338	251,347	261,685 (260,793, 262,577)	−4%
Coaches + Capacity	6419	251,748	258,167 (257,275, 259,059)	−5%
Capacity + Navigators + Coaches	10,845	230,448	241,293 (240,401, 242,185)	−11%

Abbreviations

*CI* Confidence interval

*MOT* Mandatory opioid testing during prenatal care

*NS* Not significant at the 95% level

The results were similar from the perspective of overall system incremental costs, which ranged from 3 to 24% cost reduction from baseline, as shown in Table 2. It is interesting to note, however, that overdose death costs significantly increased from baseline using the intervention of mandatory opioid testing. This cost increase was offset by the decrease in other costs, particularly the cost of living with OUD as more women end up going through treatment.

**Table 2:** Average total system incremental cost differences compared to baseline for four interventions and their combinations (all differences are significant unless shown otherwise)

Intervention	Total cost by category ($)				Overall Total (95% CI)	Percent Difference from Baseline
	Medicaid	Overdose Death	Living with OUD	Special Education		
Baseline	271,648	254,888	82,583	22,867	631,986 (625,924, 638,048)	
MOT	250,419	262,233	60,872	20,086	593,610 (587,548, 599,672)	−6%
Patient Navigators	262,090	257,922	73,095	21,632	614,739 (608,677, 620,800)	−3%
Capacity Increase	270,169	253,869	80,790	22,654	627,482 (621,420, 633,543)	−1% (NS)
Peer Recovery Coaches	262,194	251,909	81,255	22,024	617,382 (611,321, 623,444)	−2%
Navigators + Coaches	244,143	241,284	70,635	20,064	576,126 (570,065, 582,188)	−9%
MOT + Navigators	241,843	260,537	51,588	18,909	572,877 (566,816, 578,939)	−9%
MOT + Capacity	247,894	252,608	58,892	19,792	579,186 (573,125, 585,248)	−8%
MOT + Coaches	220,872	230,673	56,888	17,488	525,921 (519,859, 531,982)	−17%
MOT *+* Navigators + Coaches	201,212	218,299	45,713	15,360	480,584 (474,523, 486,646)	−24%
Navigators + Capacity	261,685	252,737	72,111	21,529	608,062 (602,001, 614,124)	−4%
Coaches + Capacity	258,167	245,379	78,789	21,581	603,916 (597,854, 609,978)	−4%
Capacity + Navigators + Coaches	241,293	240,841	69,317	19,793	571,243 (565,182, 577,306)	−10%

Abbreviations

*CI* Confidence interval

*MOT* Mandatory opioid testing during prenatal care

*NS* Not significant at the 95% level

The effect size of the four interventions and their combinations are shown in Table 3 for starting treatment, completing treatment, and NAS cases. The baseline population included 239,200 Medicaid-enrolled pregnant women and yielded yielded 15.4 cases per 1000 births (3697/239,200). From the period covered in the Indiana Medicaid claims data, there were 9417 babies diagnosed with NAS, which corresponds to 15.2 cases per 1000 births. The base case estimate also compares closely to the national estimate of 14.4 cases per 1000 births in 2014 [2]. In addition, the fraction of Indiana Medicaid-enrolled pregnant women with OUD that received treatment to those that did not receive treatment was within 4.8% of the equivalent estimate for baseline. The number of NAS cases significantly reduced by 1% for capacity increase to 32% for MOT with peer recovery coaching and peer navigators.

**Table 3:** Effect size compared to baseline for four interventions and their combinations (all differences are significant unless shown otherwise) for a population of 239,200 Medicaid enrolled pregnant women

Intervention	Started Treatment		Completed Treatement		NAS Cases	
	Average Number (95% CI)	% Difference from Baseline	Average Number (95% CI)	% Difference from Baseline	Average Number (95% CI)	% Difference from Baseline
Baseline	570.7 (563.35–578.05)		418.34 (411.83–424.85)		3697.32 (3685.95–3708.69)	
MOT	1819.17 (1811.82–1826.52)	219%	1329.48 (1322.97–1335.99)	218%	3255.66 (3244.29–3267.03)	−12%
Patient Navigators	1110.22 (1102.87–1117.57)	95%	807.92 (801.41–814.43)	93%	3505.66 (3494.29–3517.03)	−5%
Capacity Increase	679.2 (671.85–686.55)	19%	497.38 (490.87–503.89)	19%	3660.91 (3649.54–3672.28)	−1%
Peer Recovery Coaches	567.83 (560.48–575.18)	− 1% (NS)	473.89 (467.38–480.40)	13%	3576.29 (3564.92–3587.66)	−3%
Navigators + Coaches	1110.8 (1103.45–1118.15)	95%	928.38 (921.87–934.89)	122%	3234.96 (3223.59–3246.33)	−13%
MOT + Navigators	2352.22 (2344.87–2359.57)	312%	1715.83 (1709.32–1722.34)	310%	3061.97 (3050.60–3073.34)	−17%
MOT + Capacity	1924.75 (1917.40–1932.10)	237%	1408.04 (1401.53–1414.55)	237%	3207.71 (3196.34–3219.08)	−13%
MOT + Coaches	1810.38 (1803.03–1817.73)	217%	1513.61 (1507.10–1520.12)	262%	2818.27 (2806.90–2829.64)	−24%
MOT *+* Navigators + Coaches	2359.01 (2351.66–2366.36)	313%	1968.82 (1962.31–1975.33)	371%	2506.03 (2494.66–2517.40)	−32%
Navigators + Capacity	1174.2 (1166.85–1181.55)	106%	857.9 (851.45–864.47)	105%	3485.22 (3473.85–3496.59)	−6%
Coaches + Capacity	692.3 (684.95–699.65)	21%	577.8 (571.30–584.32)	38%	3481.96 (3470.59–3493.33)	−6%
Capacity + Navigators + Coaches	1174.44 (1167.09–1181.79)	106%	982.05 (975.54–988.56)	135%	3197.85 (3186.48–3209.22)	−14%

Abbreviations

*CI* Confidence interval

*MOT* Mandatory opioid testing during prenatal care

*NS* Not significant at the 95% level

## Discussion

NAS is a significant healthcare issue with potentially long-term consequences. Due to the complexity of the care process, it is difficult to quantify the impact of interventions designed to reduce its effect. Through the use of Monte Carlo simulation, we showed that a number of interventions can significantly decrease total average incremental costs both from a direct Medicaid cost perspective and overall system cost perspective. Effective implementation of these interventions, however, requires the involvement and coordination of several stakeholders.

The single intervention that led to the largest percent reduction in incremental costs was MOT. However, this intervention is not without controversy [11]. Roughly 70% of pregnant women with OUD are afraid of been identified as substance users [12] and many of those not in OUD treatment have stated they would drop out of prenatal treatment if they believe they will be required to take a drug test [13]. Further, we find that mandatory testing would increase overdose death costs. When designing such an intervention protocol, therefore, it may be important to involve child services and ensure women that there would not be punitive consequences.

There are several limitations to our study. First, we did not consider either the incremental cost of a miscarriage or of child morbidity (e.g., low birthweight, breathing and feeding problems) due to NAS other than special education needs. However, for all interventions other than mandatory opioid testing, these would only increase the system average cost at baseline, so our estimates are conservative. Second, a clear limitation of our model validation approach is that not all pregnant women with OUD would be recognized by the healthcare system as having OUD. Therefore, our assumption that the ratio of those seeking treatment to those identified as having OUD while pregnant is equivalent to the ratio of those seeking treatment to those who have OUD while pregnant but not coded as such, may not be accurate. In addition, as our parameters came from the literature, we likely did not capture all potential interactions due to multiple interventions. We also did not consider all potential interventions, which could include rooming in with family and kangaroo care. Finally, our model validation was limited to the baseline case.

## Conclusions

This study showed that a number of NAS interventions can significantly decrease total average incremental costs both from a direct Medicaid cost perspective and overall healthcare system cost perspective. We estimated that MOT would reduce hospital-associated costs for NAS by 6% from baseline and MOT with peer recovery coaching and peer navigators would reduce Medicaid-associated costs by 24% from baseline. However, MOT implementation is not without controversy. The intervention without MOT use with the largest estimated incremental cost reduction was the combination of patient navigators plus peer recovery coaches. Note that under that stated conditions, provider capacity did not a significantly reduce costs.

Although the interventions other than capacity are commonly available, we do assume that the provider would have access to navigators and coaches. Many providers have developed peer navigator programs, but most peer coaching programs are external to the provider, and that relationship would need to be developed and integrated. Conditioned on sufficient access to peer recovery coaches, however, it is not difficult to combine MOT, peer navigators, and peer recovery coaches as they can be independently administered. These findings can help both providers and Medicaid-policymakers guidance on NAS-based investments.

## Supplementary Information

**Additional file 1.** Appendix 1 References. Fig. 1. Probability tree for community-based interventions. Fig. 2. Probability tree considering mandatory opioids testing. Fig. 3. Probability tree considering use of navigators. Fig. 4. Probability tree considering capacity expansion. Fig. 5. Probability tree considering use of peer coaches. Table 1. Transitions probabilities among states and references.

**Additional file 2.** Appendix 2 References. 1. NAS treatment calculation. 2. Death cost calculations. 3. Cost for treatements.

## Data Availability

Data used in the analysis as well as all programs used for the analysis may be obtained by contacting the contacting the corresponding author on reasonable request.

## Abbreviations

CI: Confidence interval
MAT: Me
MOT: Mandatory opioid testing during prenatal care
NAS: Neonatal abstinence syndrome
NS: Not significant at the 95% level
OUD: Opioid use disorder
SD: Standard deviation
