# Supplementary material for: Estimating the impact of neonatal abstinence system interventions on Medicaid: an incremental cost analysis
Source: Subst Abuse Treat Prev Policy. 2021 Dec 20;16:91. doi: 10.1186/s13011-021-00427-1 (PMC8691068; doi:10.1186/s13011-021-00427-1)
Supplement: Supplementary file 1 — Additional file 1. Appendix 1 References. Fig. 1. Probability tree for community-based interventions. Fig. 2. Probability tree considering mandatory opioids testing. Fig. 3. Probability tree considering use of navigators. Fig. 4. Probability tree considering capacity expansion. Fig. 5. Probability tree considering use of peer coaches. Table 1. Transitions probabilities among states and references. [file 13011_2021_427_MOESM1_ESM.docx]

Appendix 1. probability trees instances.


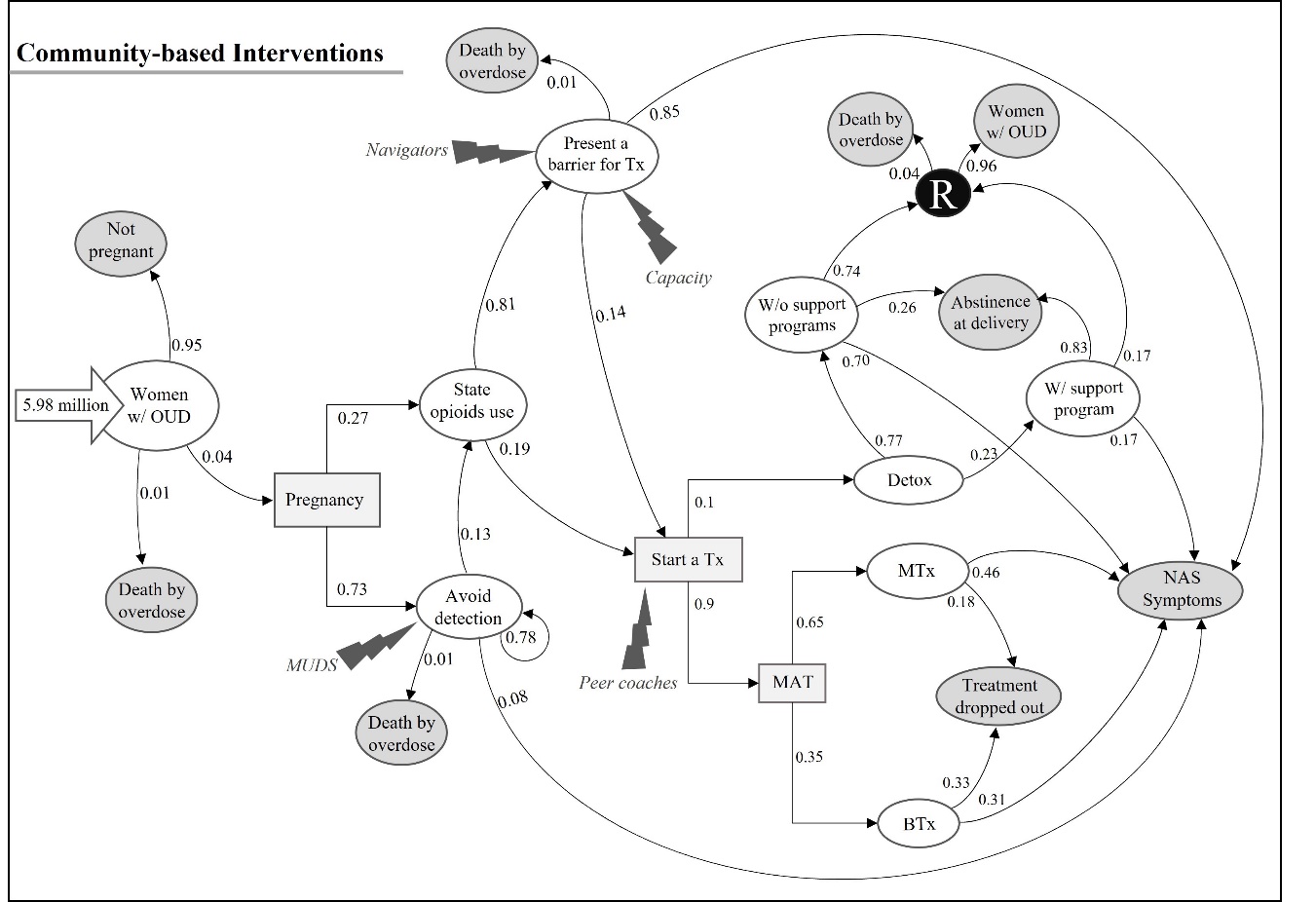


Figure 1. Probability tree for community-based interventions.

Changes considered by intervention


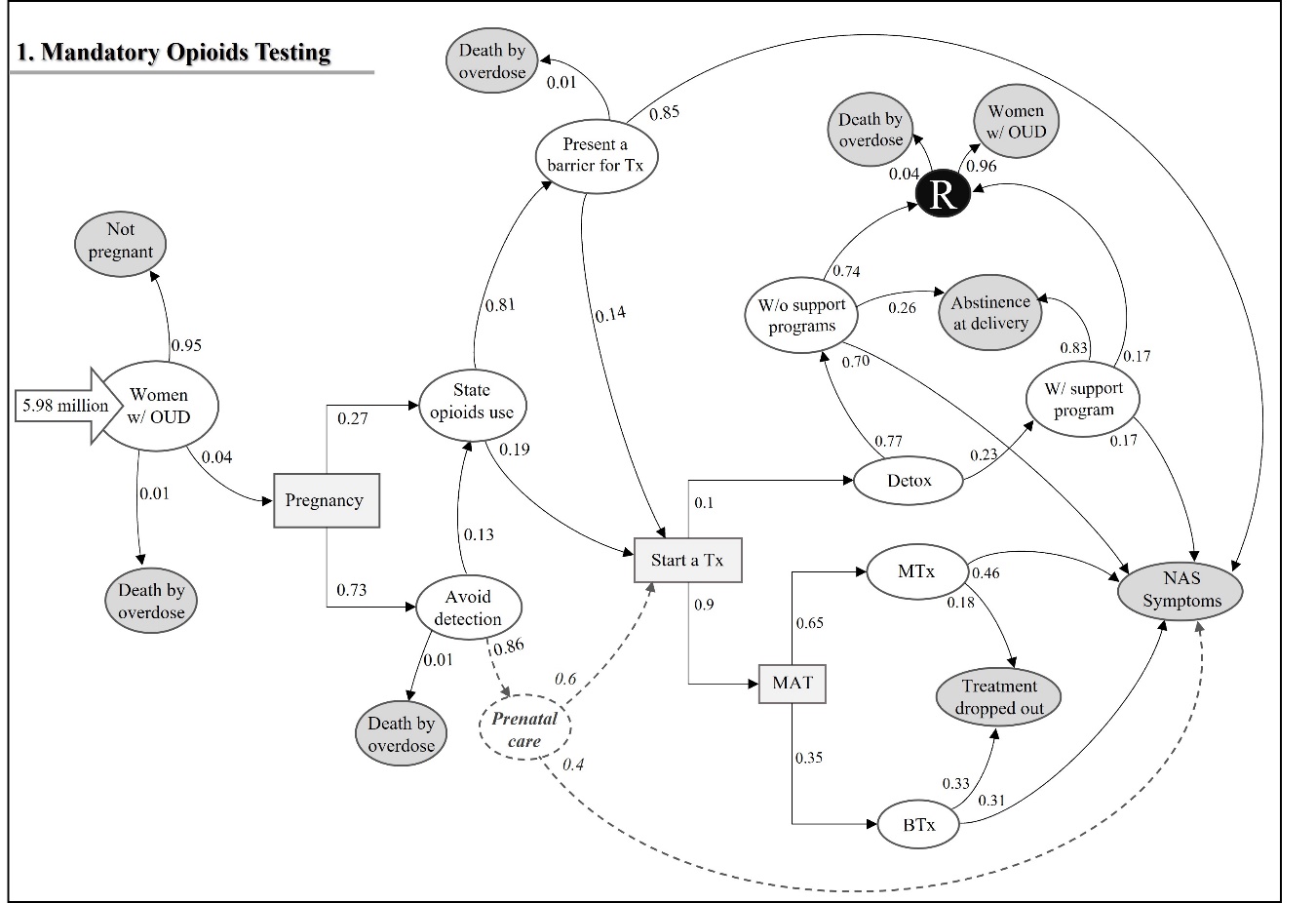


Figure 2. Probability tree considering mandatory opioids testing.


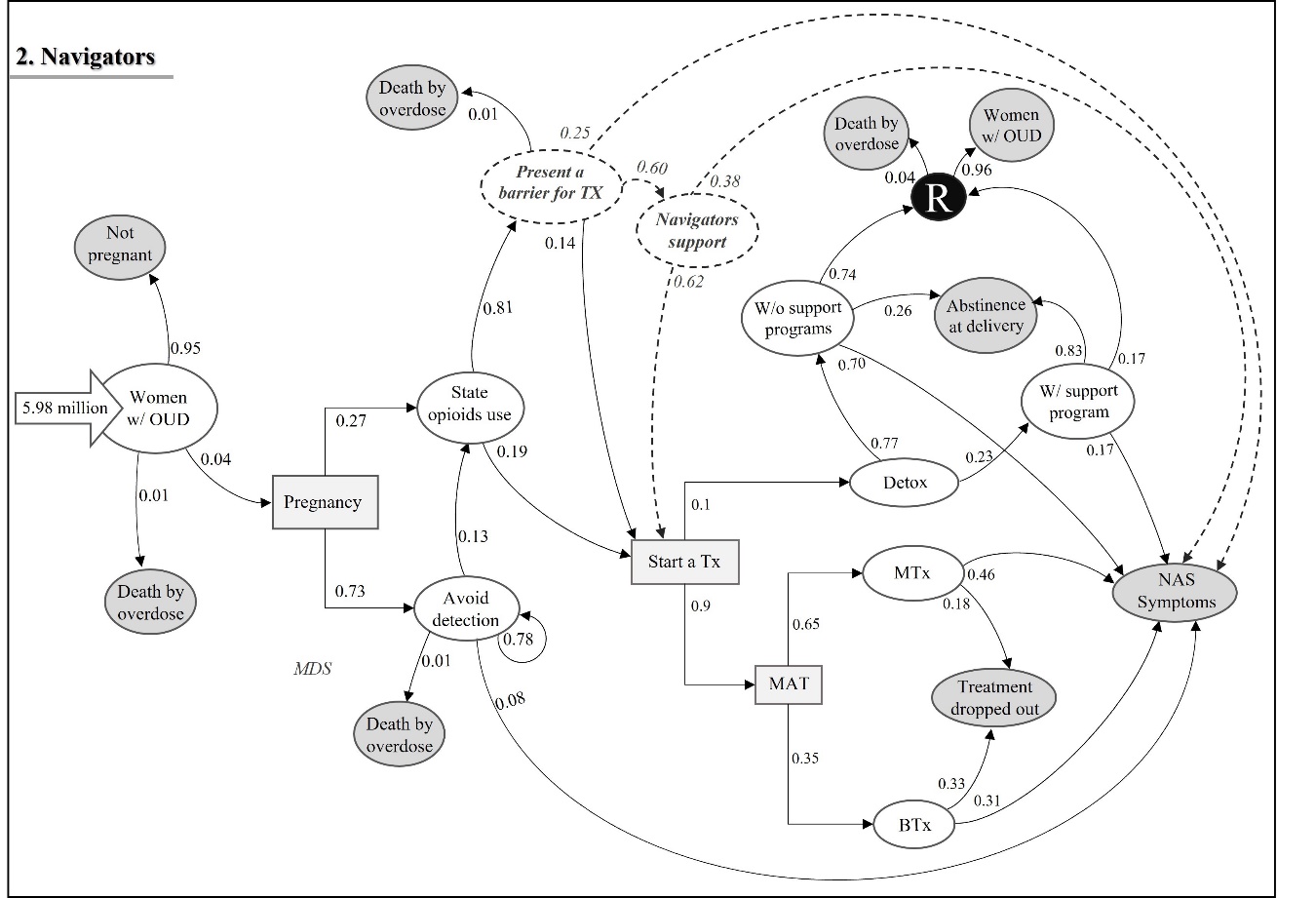


Figure 3. Probability tree considering use of navigators.


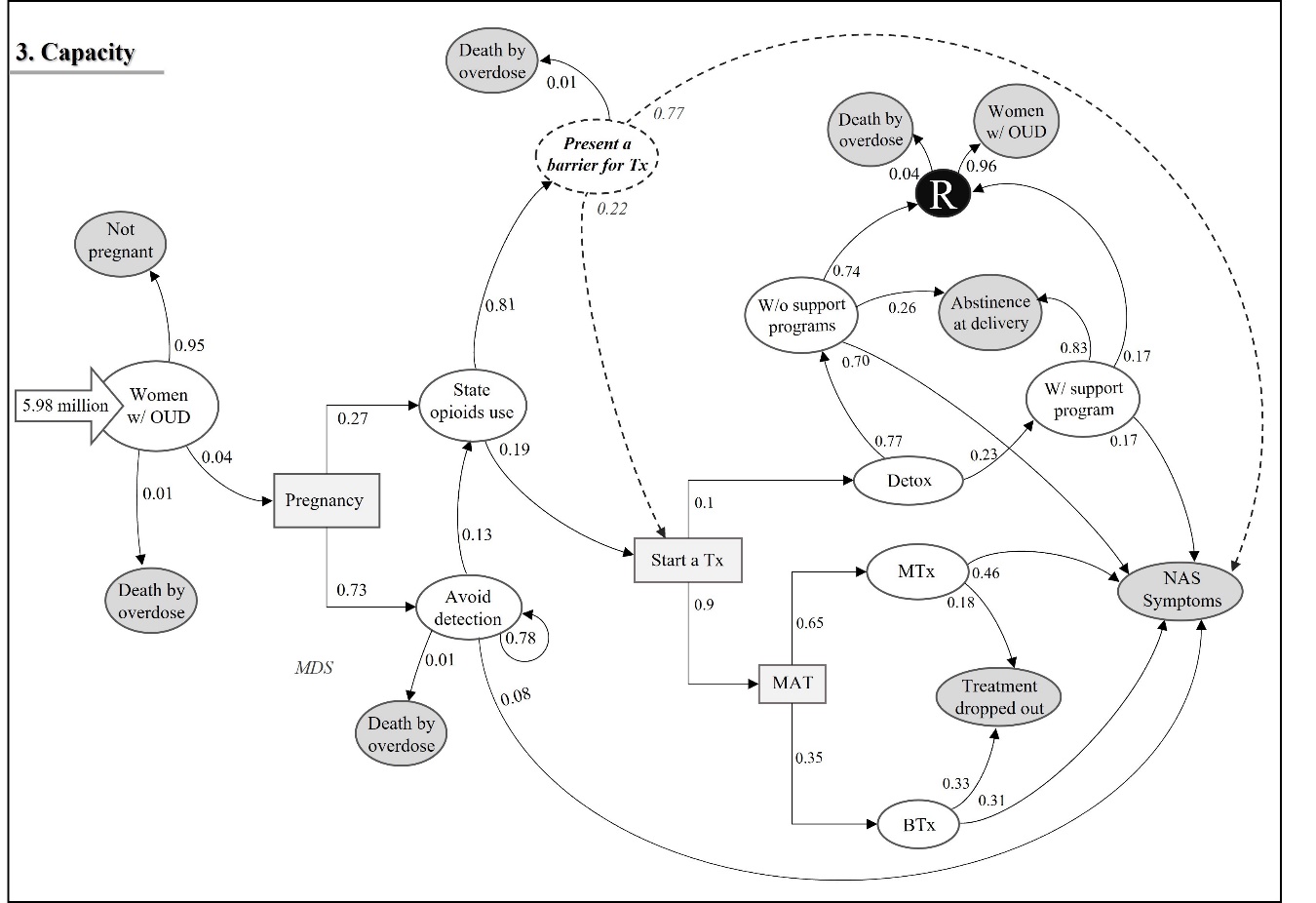


Figure 4. Probability tree considering capacity expansion.


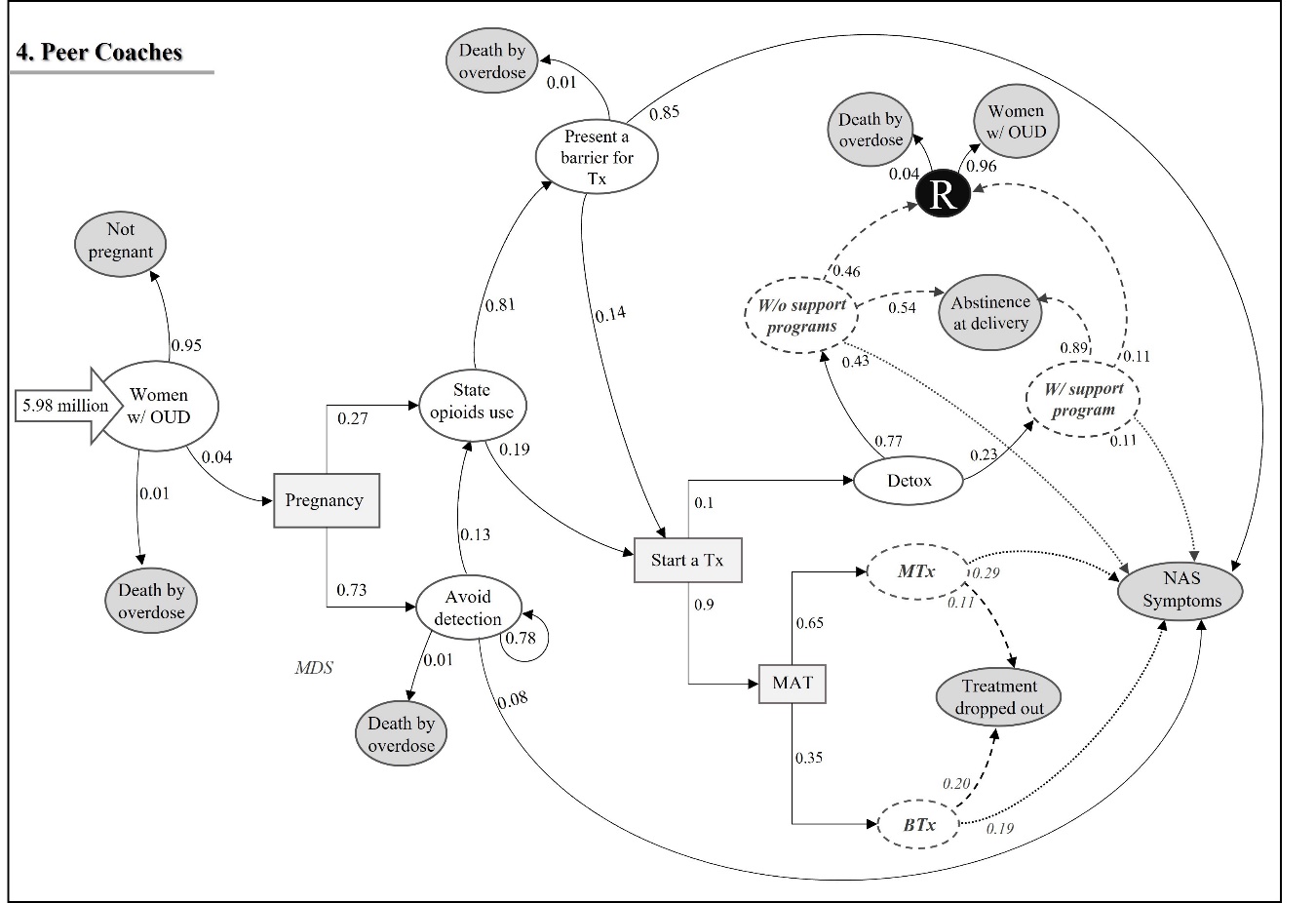


Figure 5. Probability tree considering use of peer coaches.

Table 1. Transitions probabilities among states and references.

| **State transition** | | **Transition Probability** | **Reference** |
| --- | --- | --- | --- |
| **Actual** | **Possible** |  |  |
| Women with OUD | Not pregnant | 0.95 | SAHMSA [1] |
|  | Death by OD | 0.01 | SAHMSA [1] |
|  | Pregnancy | 0.04 | SAHMSA [1] |
| Pregnancy | State Use | 0.27 | Stone [2] |
|  | Avoid Detection | 0.73 | Stone [2] |
| State Use | Start Tx | 0.19 | Jackson & Shannon [3] |
|  | Present Barrier | 0.81 | Jackson & Shannon [3] |
| Avoid Detection | State Use | 0.13 | Stone [2] |
|  | Keep avoiding | 0.86 | Stone [2] |
|  | Death by OD | 0.01 | SAHMSA [1] |
| Present barriers | Death by OD | 0.01 | SAHMSA [1] |
|  | NAS Symptoms | 0.85 | Ranji, et al. [4], Brogly et al. [5] |
|  | Start Tx | 0.14 | Ranji, et al. [4] |
| Start Tx | Detox | 0.1 | Kotelchuck et al. [6] |
|  | MAT | 0.9 | Kotelchuck et al. [6] |
| Detox | With support | 0.23 | Bell et al. [7] |
|  | No support | 0.77 | Bell et al. [7] |
| Detox With support | Relapse | 0.17 | Bell et al. [7] |
|  | Abstinence | 0.83 | Bell et al. [7] |
|  | NAS Symptoms | 0.17 | Bell et al. [7] |
|  | No NAS Symptoms | 0.83 | Bell et al. [7] |
| Detox No support | Relapse | 0.74 | Bell et al. [7] |
|  | Abstinence | 0.26 | Bell et al. [7] |
|  | NAS Symptoms | 0.7 | Bell et al. [7] |
|  | No NAS Symptoms | 0.3 | Bell et al. [7] |
| Relapse | Death by OD | 0.04 | Gossop et al. [8] |
|  | Women with OUD | 0.96 | Gossop et al. [8] |
| MAT | Methadone | 0.65 | Welle-Strand et al. [9], Black et al. [10], Lacroix, et al. [11] |
|  | Buprenorphine | 0.35 | Welle-Strand et al. [21], Black et al. [10], Lacroix, et al. [11] |
| Methadone | Tx dropped out | 0.18 | Jones et al. [12] |
|  | Tx continued | 0.82 | Jones et al. [12] |
|  | NAS Symptoms | 0.46 | Jones et al. [12] |
|  | No NAS Symptoms | 0.54 | Jones et al. [12] |
| Buprenorphine | Tx dropped out | 0.33 | Jones et al. [12] |
|  | Tx continued | 0.67 | Jones et al. [12] |
|  | NAS Symptoms | 0.31 | Jones et al. [12] |
|  | No NAS Symptoms | 0.69 | Jones et al. [12] |

**References**

1. Substance Abuse and Mental Health Services Administration (SAMHSA). Results from the 2016 National Survey on Drug Use and Health: Detailed Tables. Rockville, MD: Center for Behavioral Health Statistics and Quality*.* 2017.
2. Stone R. Pregnant women and substance use: fear, stigma, and barriers to care. Health & Justice*.* 2015;3(1):2.
3. Jackson A, Shannon L. Barriers to receiving substance abuse treatment among rural pregnant women in Kentucky. Maternal and Child Health Journal. 2012;16(9):1762-1770.
4. Ranji U, Rosenzweig C, Gomez I. Salganicoff A. Overview: 2017 Kaiser Women’s Health Survey [Internet]. Kaiser Family Foundation; 2018 Mar 13 [cited 2019 Jul]. Available from: <https://www.kff.org/report-section/methodology-2017-kaiser-womens-health-survey/>.
5. Brogly SB, Link K, Newman A. Barriers to Treatment for Substance Use Disorders among Women with Children. Canadian Journal of Addiction. 2018;9(3):18-22.
6. Kotelchuck M, Cheng ER, Belanoff C, Cabral HJ, Babakhanlou-Chase H, Derrington T, Diop H, Evans SR, Bernstein J. The prevalence and impact of substance use disorder and treatment on maternal obstetric experiences and birth outcomes among singleton deliveries in Massachusetts. Maternal and Child Health Journal. 2017;21(4):893-902.
7. Bell J, Towers CV, Hennessy MD, Heitzman C, Smith B, Chattin K. Detoxification from opiate drugs during pregnancy. American Journal of Obstetrics and Gynecology. 2016;215(3):374-e1.
8. Gossop M, Green L, Phillips G, Bradley B. Factors predicting outcome among opiate addicts after treatment. British Journal of Clinical Psychology*.* 1990;29(2):209-216.
9. Welle-Strand GK, Skurtveit S, Jones HE, Waal H, Bakstad B, Bjarkø L, Ravndal E, Neonatal outcomes following in utero exposure to methadone or buprenorphine: a National Cohort Study of opioid-agonist treatment of Pregnant Women in Norway from 1996 to 2009. Drug and Alcohol Dependence. 2013;127(1-3):200-206.
10. Black KI, Stephens C, Haber PS, Lintzeris N. Unplanned pregnancy and contraceptive use in women attending drug treatment services. Australian and New Zealand Journal of Obstetrics and Gynaecology, 2012;52(2):146-150.
11. Lacroix I, Berrebi A, Garipuy D, Schmitt L, Hammou Y, Chaumerliac C, Lapeyre-Mestre M, Montastruc JL, Damase-Michel C. Buprenorphine versus methadone in pregnant opioid-dependent women: a prospective multicenter study. European Journal of Clinical Pharmacology. 2011;67(10):1053.
12. Jones HE, Kaltenbach K, Heil SH, Stine SM, Coyle MG, Arria AM, O’Grady KE, Selby P, Martin PR, Fischer G. Neonatal abstinence syndrome after methadone or buprenorphine exposure. New England Journal of Medicine. 2010; 363(24): 2320-2331.
