# Supplementary material for: Estimating the impact of neonatal abstinence system interventions on Medicaid: an incremental cost analysis
Source: Subst Abuse Treat Prev Policy. 2021 Dec 20;16:91. doi: 10.1186/s13011-021-00427-1 (PMC8691068; doi:10.1186/s13011-021-00427-1)
Supplement: Supplementary file 2 — Additional file 2. Appendix 2 References. 1. NAS treatment calculation. 2. Death cost calculations. 3. Cost for treatements. [file 13011_2021_427_MOESM2_ESM.docx]

**Appendix 2. Costs Calculations**

1. **NAS treatment calculation**

According to Milliren et al. (2018) the average length of stay of a baby for NAS treatment is 16.1 days (SD: 13.3). Considering this the range is 2.8 – 29.4 days. Per baby that reaches NAS state in the tree, random number between this range is calculated.

Treatment cost estimation is generated by days of treatment estimation times average cost per day.

Average cost per day is calculated based on the average cost for NAS treatment (US$ 38,687) and the average of length of stay (16.1) presented by Milleren et al. (2017). Average cost per day = US$ 2,400.

The estimation per student who receives special education is US$ 15,341 per year. (Morgan and Wang, 2019). Special education for is considered for 20% of children that presented NAS symptoms.

1. **Death cost calculations**

The cost considered is the present value of lifetime productivity by age presented by Grosse et al. (2016). Age range considered is 16-40 years old. Per woman, a number is generated to represent the age and the cost is assigned based on the number:

| Age (years) | Present value of lifetime productivity |
| --- | --- |
| X < 20 | $ 1,913,589.00 |
| 20 < X < 30 | $ 1,977,803.00 |
| 30 < X < 40 | $ 1,744,962.00 |

1. **Cost for treatments**

According to the Office of the secretary, Department of defense (2016) medication for supervised detoxification is $ 18 for methadone and $ 22.5 for buprenorphine (USD per day).

1. *DETOX*

We used the average of these costs [(18 + 22.5) / 2] = $ 20.25 USD per day. For detoxification without support, considers the treatment psychosocial support just during the treatment 30 days (US$ 607.5). Detox with support considers this help for 90 days (US$ 1,822.5).

1. MEDICAL ASSISTET TREATMENTS (MAT).

A woman could be in treatment 1 day or the entire pregnancy (280 days). MAT cost calculations:

1. If the treatment goes to the complete pregnancy (*Assumes it was for the hole pregnancy):

- Total cost for a finished Methadone treatment = US$ 5,040
- Total cost for a finished buprenorphine treatment = US$ 6,300

1. If the treatment is not finished, a random number is generated for the days of treatment between 1.5 and 275.

The number of days times the cost of the day will be the cost for not finished treatment.

**Cost for living with OUD**

A diagnosed abuser had, on average, excess annual healthcare costs of US$ 10,627 and US$ 1,244 in excess annual work-loss costs. (Rice et al. 2014). Total extra healthcare cost for diagnosed OUD persons = $ 11,871

**Discounting**

As the time horizon was one year, costs were not discounted.

**References**

# Department of defense (2016). *TRICARE; Mental Health and Substance Use Disorder Treatment. The Daily Journal of the United States Government. 09/02/2016.* https://www.federalregister.gov/documents/2016/09/02/2016-21125/tricare-mental-health-and-substance-use-disorder-treatment#citation-2-p61074

Grosse, S. D., Krueger, K. V., & Pike, J. (2019). Estimated annual and lifetime labor productivity in the United States, 2016: implications for economic evaluations. *Journal of medical economics*, *22*(6), 501-508.

Milliren, C. E., Gupta, M., Graham, D. A., Melvin, P., Jorina, M., & Ozonoff, A. (2018). Hospital variation in neonatal abstinence syndrome incidence, treatment modalities, resource use, and costs across pediatric hospitals in the United States, 2013 to 2016. *Hospital pediatrics*, *8*(1), 15-20.

Morgan, P. L., & Wang, Y. (2019). The opioid epidemic, neonatal abstinence syndrome and estimated costs for special education services. *The American journal of managed care*, *25*(13 Suppl), S264-S269.

Rice, J. B., Kirson, N. Y., Shei, A., Cummings, A. K. G., Bodnar, K., Birnbaum, H. G., & Ben-Joseph, R. (2014). Estimating the costs of opioid abuse and dependence from an employer perspective: a retrospective analysis using administrative claims data. *Applied health economics and health policy*, *12*(4), 435-446.
